# Development and relative validation of a short food frequency questionnaire for assessing dietary intakes of non-alcoholic fatty liver disease patients

**DOI:** 10.1007/s00394-019-01926-5

**Published:** 2019-02-25

**Authors:** Carla Bredin, Sara Naimimohasses, Suzanne Norris, Ciara Wright, Neil Hancock, Kathryn Hart, J. Bernadette Moore

**Affiliations:** 1 Department of Nutritional Sciences, University of Surrey, Guildford, GU2 7XH Surrey UK; 2 Hepatology Department, St James’ Hospital, Dublin, Ireland; 3 Department of Clinical Medicine, Trinity College Dublin, Dublin, Ireland; 4 Glenville Nutrition, Dublin, Ireland; 5 School of Food Science and Nutrition, University of Leeds, Leeds, LS2 9JT UK

**Keywords:** Short food frequency questionnaire, Non-alcoholic fatty liver disease, Diet, Nutrition

## Abstract

**Purpose:**

This work aimed to design and validate a novel short food frequency questionnaire (SFFQ) to assess habitual intakes of food items related to non-alcoholic fatty liver disease (NAFLD) in a cohort of European patients.

**Methods:**

A 48-item SFFQ was created, with questions from existing FFQs and expert knowledge, emphasizing foods and nutrients implicated in NAFLD pathogenesis. Consenting, fibroscan-diagnosed, NAFLD patients completed the SFFQ during a short interview and were asked to complete a 4-day diet diary (4DDD) at home for return by mail. Nutritional intakes were assessed utilizing the myfood24™ food composition dataset and estimated energy requirements (EER) were calculated using sex-, age- and weight-specific equations. Agreement between the dietary instruments was assessed by Spearman correlations and Bland Altman analysis.

**Results:**

Fifty-five patients completed both the SFFQ and the 4DDD within 30 weeks; 42 (76%) were diagnosed with simple steatosis, whereas 13 (24%) had biopsy-proven steatohepatitis; the majority were overweight or obese, with a median (25th; 75th percentile) BMI of 33.2 kg/m^2^ (29.3; 36.0). Reported energy intakes were well below EER with a median intake of 73% of requirements, suggesting widespread under-reporting. Significant correlations were observed between sugar (*r* = 0.408, *P* = 0.002), fat (*r* = 0.44, *P* = 0.001), fruits (*r* = 0.51, *P* = 0.0001) and vegetables (*r* = 0.40, *P* = 0.0024) measurements by the SFFQ and 4DDD. Bland Altman plots with regression analysis demonstrated broad comparability with the 4DDD for intakes of fat (bias − 13.8 g/day) and sugar (bias  + 12.9 g/day).

**Conclusions:**

A novel SFFQ designed to be minimally burdensome to participants was effective at assessing dietary intakes in NAFLD patients.

**Electronic supplementary material:**

The online version of this article (10.1007/s00394-019-01926-5) contains supplementary material, which is available to authorized users.

## Introduction

Non-alcoholic fatty liver disease (NAFLD) is currently the leading cause of liver disease in the developed world with incidence corresponding to increasing obesity and diabetes trends worldwide [1]. Characterised by excessive fat accumulation in the liver, NAFLD diagnosis is based on the presence of steatosis in > 5% of hepatocytes and the exclusion of: significant alcohol consumption (> 30 g/day for men; >20 g/day for women); use of hepatotoxic medications such as methotrexate, tamoxifen or steroids; and other liver diseases such as Hepatitis C viral infection, haemochromatosis, or Wilson’s Disease [2]. Histologically, NAFLD may range from simple steatosis (NAFL), where there is fatty infiltration but no evidence of hepatocellular injury, to steatohepatitis (NASH), where there is evidence of inflammation and ballooning, with or without fibrosis [3]. Closely associated with obesity and co-morbidities such as type 2 diabetes, and cardiovascular disease, the prevalence of NAFLD varies across the world with estimates of 17–46%, depending on diagnostic method, age and gender of the patient [1]. Given the similarities in aetiology, NAFLD is commonly described as the hepatic manifestation of the metabolic syndrome [4]. In particular, hyper-energetic diets, containing high levels of saturated fat, refined carbohydrates and sugar sweetened beverages, are strongly implicated in NAFLD pathogenesis. Currently, in the absence of existing first-line pharmacotherapy, dietary and lifestyle changes aimed at weight loss are fundamental to clinical management guidelines [2, 3, 5]. Significant reduction in steatosis and hepatic markers of NAFLD has generally been observed with a weight loss of 5–10% [6].

In addition to excess energy, evidence is accumulating that dietary quality and specific dietary components may play a distinct role in NAFLD pathogenesis. Consistently, a ‘Western style’ diet, with high intakes of meat, saturated fat and sugar, and low intakes of fruits, vegetables, fibre and omega 3 polyunsaturated fats has been associated with NAFLD disease severity [7]. Given the challenge of sustaining weight loss for most people [8], altering dietary composition in the absence of caloric restriction may be more feasible and may improve steatosis and metabolic dysfunction in NAFLD [9]. Notably, in a recent trial examining the effects of isocaloric, ad libitum Mediterranean and low-fat diets on steatosis and cardiometabolic risk factors, both diets reduced liver fat significantly after 12-weeks to a similar degree, 25–32%, measured by magnetic resonance spectroscopy. Interestingly, while the intervention was not designed for weight loss, and there was no difference in the caloric intakes measured at baseline and 12-weeks, both groups lost a small (2%) amount of weight lower than that typically associated with NAFLD improvement [10]. While no differences between the dietary groups were observed in the reductions of liver fat and body weight, improvements in total cholesterol, plasma triglycerides, and HbA1c levels were observed in the Mediterranean diet group; in line with the body of evidence suggesting this dietary pattern reduces metabolic risk factors and cardiovascular disease risk [11–13].

Dietary assessment for nutrition research can be divided into two distinct groups that either assess quantitatively short-term, daily nutrient intake through dietary records and interviewer-aided 24-h dietary recalls; or assess long-term habitual food consumption through food frequency questionnaires (FFQ). Respondent-led dietary records, such as 4-day diet diaries (4DDD), require a level of participant burden that has been well-characterised as a limitation of use, alongside other limitations such as reactivity bias (change in behaviour due to monitoring of behaviour [14]), errors in estimation of portion sizes, and non-compliance [15]. Similar limitations exist for FFQs, which while typically used for large population studies, may be extensive and time-consuming, with significant participant burden, such as the 198-item FFQ from the European Prospective Investigation into Cancer and Nutrition (EPIC) study [16]. FFQs are routinely adapted, often for concision, and typically validated for a specific population being studied. For example, the 217-item FFQ created for the UK Women’s cohort study [17] was utilised by Cleghorn and colleagues [18] to validate an abridged, 20-item short FFFQ (SFFQ) for the purpose of assessing dietary quality conveniently in UK adults, irrespective of gender.

While the advantages of FFQs include ease of administration, particularly in settings lacking formal dietetic support, and the ability to embed questions pertaining to portion size [15], limitations to these exist. Studies utilising doubly labelled water to accurately measure total energy expenditure and/or urinary nitrogen as a biomarker of protein intake, have shown FFQs (and indeed also multiple 24 h recalls) severely underestimate absolute intakes of energy and protein [19, 20]. Although often polarised debates have ensued about the utility of these instruments in nutritional epidemiological research, nonetheless dietary data derived from these imperfect instruments have proven to be useful in addressing important research and public policy questions [21]. The administration of multiple short term dietary assessment instruments is accepted as providing more valid dietary information; and new technologies, including web-based and smartphone applications, now permit low-cost dietary assessment at scale [22]. However, doubly labelled water studies suggest caution is still warranted for energy estimates from these [23]. The use of electronic tools in low resource settings and in low literacy populations is still limited [24].

In the context of clinical care, dietary assessment is used alongside biochemical, physical, and anthropometric measurements [15]. Dietary assessment should be used to provide individualised dietary advice and to evaluate the success of interventions to improve diet and morbidity associated with chronic conditions such as obesity, diabetes and NAFLD. However, many primary care and tertiary referral settings lack resources for specialist dietetic support. Brief dietary assessment tools, responsive to dietary changes, can allow health professionals with minimal nutrition knowledge to quickly identify areas of concern and help set and monitor food-based dietary goals for their patients [25]. While several disease-specific tools exist and have been used in clinical practice, the majority of these have been developed and evaluated in the US for dietary management of cardiovascular disease and type 2 diabetes [25]; to our knowledge no such instrument has been developed for NAFLD patients or for a European population. Therefore, the aim of this research was to develop and validate a short food frequency questionnaire (SFFQ) capable of assessing foods and drinks, associated with obesity and NAFLD, consumed during a ‘typical’ week over the past month, which could be used in settings lacking specialist dietetic support in an efficient and cost-effective manner. In addition, we aimed to characterise, for the first time, the dietary intakes of Irish patients with a diagnosis of NAFLD in tertiary care using the 4 day diet diary (4DDD) reference method.

## Materials and methods

### Study participants

Given the aim of developing a SFFQ for use with NAFLD patients in tertiary care, 55 consenting participants over the age of 18, with a Fibroscan and/or liver biopsy-based diagnosis of NAFLD, were recruited from the Hepatology Department in St James’ Hospital, Dublin, Ireland between January and September 2017 during their routine clinic visits. Ethical approval was obtained from both St James’ Hospital/Adelaide and Meath Research Ethics Committee (ref 2017-01) and the Faculty of Health and Medical Sciences Ethics Committee, University of Surrey (1271-FHMS-17); and this cross-sectional analysis was conducted in accordance with the 1964 Declaration of Helsinki and its later amendments. Exclusion criteria were: nutritional counselling in last 24 months, under the age of 18, and diagnosis of any other liver disease including hepatitis C and hepatitis B, coeliac disease, Wilson’s disease, autoimmune hepatitis or alcoholic fatty liver disease. Demographics, anthropometric and other related clinical data were recorded as a part of routine clinical care.

### Short food frequency questionnaire (SFFQ; test method)

A semi-quantitative SFFQ was developed using questions from previously validated instruments [16, 18], and using expert knowledge about culturally specific foods, to tailor the tool to an Irish population. The 20-item SFFQ validated by Cleghorn and workers [18] for the assessment of dietary quality, based on intakes of fruits, vegetables, oily fish, free sugars and fat, in a UK population, was used as a starting point. Additional questions were adopted from the EPIC FFQ [16] to expand on intakes of refined carbohydrates, as well as high-fat foods, and highly processed foods implicated in NAFLD pathogenesis. Frequency was based ‘on a typical week, over the last month or so’, and answers corresponded to one out of eight options, with categories ranging from ‘rarely or never’ to ‘5 + times a day for the carbohydrate group, and from ‘rarely or never’ to ‘7 + times a week’ for the protein and fat group. Additional questions related to food preparation, alcohol consumption, and food consumed outside of the home appended the SFFQ. The resulting 48-item, SFFQ (Supplementary file) was administered to each participant by the same researcher in an interview that lasted approximately 20 min. While the FFQ itself only briefly described a portion size using the phrasing from the Cleghorn SFFQ [18], ‘a portion includes: a handful of grapes, an orange, a serving of carrots, a side salad, a slice of bread, a glass of pop’, standard portion sizes from the healthy eating guidelines of the Food Safety Authority of Ireland (FSAI) [26] were explained at interview. Food groupings were derived from the, largely similar, groupings used by the Cleghorn and EPIC instruments [16, 18].

### Four-day diet diary (4DDD; reference method)

All participants were advised on how to complete a detailed written 4DDD, on 2 week days and 2 weekend days, which was returned by mail. The 8-page template consisted of 3 columns for recording type of meal (i.e. before breakfast, breakfast, before lunch, lunch, afternoon, evening meal, evening/night), time of meal, and the weights and sizes of foods/meals. Participants were encouraged to weigh foods whenever possible, and where this was not possible they were asked to give as much detail as practical on portion size using information from package information and household measures. These were converted into weights by a trained analyst using manufacturer’s information and standard portion sizes [26]. Nutrient intakes were analysed using the online, myfood24™ dietary assessment tool [27]. Diaries were inputted as four 24 h periods and mean intakes were calculated for each nutrient for each participant. Of note, the underpinning database for myfood24™ expands greatly on the UK food composition dataset [28] of ~ 3300 items by incorporating an additional > 40,000 generic and branded items [29] commonly found in UK and Irish supermarkets.

Under-reporting was calculated based on the ratio of energy intake (EI) from the 4DDDs to estimated energy requirements (EER) [30]. The EER for all participants were calculated using sex-, age- and weight-specific equations coupled with physical activity levels [31]. The physical activity level for sedentary lifestyle (1.55) was applied to all participants based on reported activity levels and lack of an objective measure of physical activity in the study design. The expected EI:EER ratio is set at 1:1, and those with intakes below and above this ratio were classified as misreporting [32].

For comparison between the two instruments, portion sizes were assigned to each food item in the SFFQ, and multiplied by the chosen frequency response [33]. These were either standard portion sizes from FSAI [26], for example 80 g of fruit or vegetables; or, derived from the average portion size of the food items taken from the myfood24™ database for the derivation of the sugar and fat estimations. Details of all calculations and food items used for nutrient derivations for the SFFQ are provided within the metadata accompanying our dataset (Supplementary file 1) and this, along with the SFFQ and patient information leaflet developed in this project, is available through an open access repository under a Creative Commons Attribution licence (CC-BY 4.0) [34].

### Statistical methods

Statistical analysis was done using GraphPad Prism v7.0 (GraphPad Software Inc, La Jolla, CA). Data were tested for normality using D’Agostino-Pearson omnibus and Shapiro–Wilk tests and results are presented as median and interquartile range (25th, 75th percentile). To compare the NAFL and NASH groups, the Mann–Whitney *U* test was used. Comparisons for categorical variables were done using Fisher’s exact test. Agreement (validity) between the SFFQ and 4DDD instruments for fruit, vegetables, total sugar, and total fat was assessed as recommended [35] by multiple statistical methods for numerical variables. These included the Wilcoxon matched-pairs signed rank test and associated Spearman’s correlation to assess median differences and effectiveness of pairing; along with Bland Altman (difference vs mean) plots with regression analysis to detect proportional differences and to indicate the direction and magnitude of the bias. In addition, Cohen’s kappa (*κ*) test was used to determine the ability of the SFFQ to rank individuals categorically based on lower (< 60 g/day, ~ 10% total energy) or higher (≥60 g/d) sugar intakes. Throughout, a *P* value < 0.05 was considered statistically significant.

## Results

Between January and September 2017, 81 consecutive patients attending the St James’ Hospital NAFLD clinic were approached for the study. Of the 55 consenting participants who completed both SFFQ and 4DDDs, 42 had NAFL and 13 had a biopsy-confirmed, diagnosis of NASH; and their characteristics are outlined in Table 1. The median (25th, 75th percentile) age of participants was 58 (52; 65). Gender was evenly split in the overall cohort, with 53% male and 47% female participants, and 96% of the cohort were overweight or obese using BMI classifications. Although there was no difference in median BMI between the NAFL, 32.2 (29.3; 36.2 kg/m^2^), and NASH groups 33.2 (29.7; 35.8 kg/m^2^), patients with NASH were more likely to have concomitant diagnoses of type 2 diabetes (77% vs. 22%; *P* = 0.0005) and hypertension (77% vs. 36%; *P* = 0.012). Patients with a diagnosis of type 2 diabetes and dyslipidaemia were typically treated with medications such as metformin and statins; as such, median HbA1c levels, 39.5 (43.3; 46.8 mmol/mol) and LDL levels, 2.4 (1.9; 2.9 mmol/L) were well-managed within this population, although triglycerides, 1.9 (1.3; 2.7 mmol/L) were somewhat elevated. NASH patients had higher Fibroscan scores, 12.9 (10.6; 14.5 kPA), than NAFL patients 6.3 (5.2; 7.8) (*P* ≤ 0.0001); and similarly had elevated liver enzymes when compared with NAFL patients; ALT (57 vs 38 IU/L; *P* = 0.0394), AST (52 vs 26 IU/L; *P* ≤ 0.0001), and GGT (97 vs 53 IU/L; *P* = 0.0178).

**Table 1:** Demographic, anthropometric and biochemical characteristics

Characteristics	Total (*n* = 55)	NAFL (*n* = 42)	NASH (*n* = 13)	*P* value^a^
Male, female *n* (%)	29, 26 (53, 47)	25, 17 (60, 40)	4, 9 (31, 69)	–
Age (years)	58 (52; 65)	55 (52; 64)	58 (50; 65)	0.9880
Weight (kg)	92 (81;103)	93.5 (84; 102.5)	84 (77; 101.8)	0.3837
Height (cm)	168 (161; 175)	168 (162; 175)	165 (155; 174)	0.3515
Body mass index (kg/m^2^)	33.2 (29.3; 36)	32.2 (29.3; 36.2)	33.2 (29.7; 35.8)	0.9228
Dyslipidaemia *n* (%)	26 (47%)	18 (43%)	8 (62%)	0.3425
Diabetes *n* (%)	19 (35%)	9 (22%)	10 (77%)	0.0005
Hypertension *n* (%)	25 (46%)	15 (36%)	10 (77%)	0.012
Triglycerides (mmol/L)	1.9 (1.3; 2.7)	1.9 (1.2; 2.7)	2.2 (1.5; 2.8)	0.3486
HDL (mmol/L)	1.3 (0.9; 1.4)	1.3 (1.0; 1.4)	1.2 (0.9; 1.3)	0.2328
LDL (mmol/L)	2.4 (1.9; 2.9)	2.5 (2.0; 2.9)	2.1 (1.5; 2.4)	0.1129
HbA1c (mmol/mol)	39.5 (43.3; 46.8)	37.0 (34.0; 43.8)	45.5 (40.8; 47.3)	0.0325
ALT (IU/L)	42 (25; 61)	38 (24; 56)	57 (31; 81)	0.0394
AST (IU/L)	31 (22; 40)	26 (22; 32)	52 (38; 83)	< 0.0001
GGT (IU/L)	60 (34; 84)	53 (33; 80)	97 (49; 190)	0.0178
FibroScan LSM (kPa)^b^	6.9 (5.6; 11.3)	6.3 (5.2; 7.8)	12.9 (10.6; 14.5)	< 0.0001
CAP Score (dB/m)^b^	316 (297; 355)	312 (292; 352)	338 (307; 358)	0.2850
NAFLD Fibrosis Score^c^	– 1.25 (– 2.68; – 0.36)	– 1.73 (– 2.79; – 0.89)	– 0.36 (– 1.25; – 0.16)	0.0551

Values are presented as number of participants (% of sample) or median (25th; 75th percentile)

*ALT* Alanine aminotransferase, *AST* aspartate aminotransferase, *BMI* body mass index, *CAP* controlled attenuation parameter, *dB/m* decibels per milliwatt, *GGT* gamma-glutamyl transferase, *HbA1c* glycated haemoglobin, *HDL* high density lipoprotein, *kPa* kilopascal, *LDL* low density lipoprotein, *LSM* liver stiffness measurement, *NAFL* non-alcoholic fatty liver, *NAFLD* non-alcoholic fatty liver disease, NASH non-alcoholic steatohepatitis

^a^Mann–Whitney *U* test performed for continuous variables (age, weight, height, BMI) and Fisher’s exact test performed for categorical variables (number of patients with and without concomitant disease in each cohort) comparing NAFL with NASH

^b^The FibroScan LSM and CAP Scores are acquired from transient elastography (specialised ultrasound) of liver and are used to noninvasively diagnose fibrosis and steatosis in the liver, respectively

^c^NAFLD fibrosis score is an algorithm based on routine clinical measurements used to predict the presence of liver fibrosis = 1.675 + 0.037 × age + 0.094 × BMI + (1.13 if diabetes) + 0.99 × (AST/ALT) − 0.014 × platelet − 0.66 × albumin

Initial dietary analysis from the 4DDD (Table 2) highlighted median energy intakes well below EER for all participants at 73% (60; 97); suggesting widespread underreporting in this cohort. While there were no differences in macronutrient consumption between NAFL and NASH patients, median intakes of saturated fatty acids (SFA), were higher than dietary guidelines at 13 (10, 16) percent total energy (%TE). Similarly, intakes of total sugars, 15 (11; 21) %TE, were higher than current guidelines worldwide [36] to keep free sugars to 5–10%TE. Although median levels of total fat at 34 (31; 41) %/TE were below Irish and EU recommendations of < 35% total energy, 47% of the cohort exceeded this. Relatedly, and perhaps unusually for a NAFLD population, reported protein intakes did not generally exceed reference intakes, with 38% of the cohort not meeting the 0.75 g protein per kilogram of body weight recommendation [37]. The vast majority of patients reported intakes of both omega 6 polyunsaturated fatty acids, (n6 PUFA) 1.2 (0.8; 2.3) %TE, and omega 3 PUFA, (n3 PUFA) 0.2 (0.1; 0.3) %TE, which were well below recommended guidelines of 1.5% and 0.5%, respectively. Dietary fibre intakes, 12.3 (8.8; 14.8) g/day, were similarly less than 50% of the recommended 25 g/day for an adult [37]. Micronutrient intakes were within adequate range of the RDA for most nutrients, including B1, B2, B3, B6, B12 and folate, as well as iron, zinc, copper and vitamin C (data not shown). Dietary vitamin D intakes were on the low-normal scale, at 1.7 (1.1; 3.0 range 0–10) µg/day; whereas median sodium intakes, 2668 (2119; 3078) mg/day exceeded the upper limit RDA (< 2500 mg/day) for many patients [37].

**Table 2:** Energy and macronutrient intakes calculated from 4-day diet diaries from patients with NAFL and NASH

	Total (*n* = 55)	NAFL (*n* = 42)	NASH (*n* = 13)	*p* value
Energy intake (kcal/day)	1625 (1390; 2090)	1621 (1389; 2102)	1699 (1398; 1999)	0.9143
EER (kcal/day)^a^	2263 (1897; 2635)	2272 (1871; 2644)	2064 (1761; 2289)	0.1973
Energy intake/requirement (%)	73 (60; 97)	72 (63; 96)	86 (59; 104)	0.6433
Macronutrients/foods				
Protein (g/day)	71.8 (66.6; 84.7)	72.2 (67.4; 86.9)	70.7 (56.9; 75.6)	0.331
Fat (g/day)	65.2 (49.1; 87.01)	62.9 (49.6; 83.2)	74 (46.1; 93.0)	0.8068
Fat (%TE)	34 (31; 41)	34 (31; 39)	35 (33; 44)	0.4989
SFA (g/day)	23.5 (17.2; 32.4)	23 (18.2; 34.1)	23.5 (11.4; 30.9)	0.3116
SFA (%TE)	13 (10; 16)	13 (11; 17)	11 (8; 14)	0.1901
n6 PUFA (g/day)	2.3 (1.3; 4.8)	2.2 (1.2; 4.4)	3.7 (1.7; 6.0)	0.1308
n6 PUFA (%TE)	1.2 (0.8; 2.3)	1.1 (0.8; 1.8)	1.5 (1.2; 3.4)	0.0944
n3 PUFA (g/day)	0.4 (0.2; 0.6)	0.4 (0.2; 0.6)	0.5 (0.3; 0.6)	0.5501
n3 PUFA (%TE)	0.2 (0.1; 0.3)	0.2 (0.1; 0.3)	0.2 (0.2; 0.4)	0.5371
MUFA (g/day)	21.4 (16.5; 32.2)	21.0 (16.8; 31.5)	23.6 (12.6; 34.2)	0.5633
MUFA (%TE)	12 (10; 13)	12 (10; 13)	13 (11; 15)	0.1526
Carbohydrate (g/day)	178 (160; 222)	183 (160; 226)	176 (161; 216)	0.7766
Carbohydrate (%TE)	45 (41; 50)	45 (44; 49)	45 (37; 52)	0.8525
Total sugars (g/day)	66.1 (45.2; 93.8)	67.7 (45.0; 96.1)	59.6 (50.0; 91.9)	0.822
Total sugars (%TE)	15 (11; 21)	16 (11; 20)	16 (13; 17)	0.8679
Dietary fibre (g/day)	12.3 (8.8; 14.8)	11.9 (8.8; 13.6)	13.8 (9.9; 15.7)	0.2261

Values are presented as median (25th; 75th percentile)

*EER* estimated energy requirement, *NAFL* non-alcoholic fatty liver, *NASH* non-alcoholic steatohepatitis, *SFA* saturated fatty acids, *MUFA* monounsaturated fatty acids, *n6* omega 6, *PA* physical activity, *PUFA* polyunsaturated fatty acids, *%TE* percent total energy

^a^Males: EER = 662 – (9.53 × age [*y*]) + PA x [(15.91 × weight [kg]) + (539.6 × height [cm])] [31]

Females: EER = 354 – (6.91 × age [y]) + PA x [(9.36 x weight [kg]) + (726 × height [cm])] [31]

Prioritising fruit, vegetables, sugars, and fat, as critical food items and nutrients implicated in NAFLD; we examined intakes assessed by the SFFQ in relation to the 4DDD (Table 3). Fruit and vegetable intakes were low in the cohort in general, equivalent to 1.5 standard portions of each as assessed by 4DDD; fruit: 125 (55–216) g/d, vegetable: 118 (86–181) g/day. Median differences were very small for fruit (– 4%) and sugar (+ 3%), but somewhat larger for fat (– 23%) and vegetables (– 52%). Spearman’s correlations between the two dietary assessment instruments were moderate and highly significant for fruit (*r* = 0.5123, *P* = 0.0001), fat (*r* = 0.4326, *P* = 0.0010) and sugar (*r* = 0.4079, *P* = 0.0020); while the vegetable measurement was more variable (*r* = 0.3983, *P* = 0.0026). The Wilcoxon matched pairs signed-rank sum test showed no significant differences in the median fruit and total sugar measurements between the SFFQ and the 4DDD; although differences were observed for fat (*P* < 0.0013) and vegetables (*P* < 0.0001; Table 3). Cohen’s kappa indicated fair agreement (*κ* = 0.347) between the SFFQ’s and 4DDD’s ranking of individuals categorically based on lower or higher sugar intakes.

**Table 3:** Comparisons of median intakes from SFFQ and 4DDD (*n* = 55) for total fat, fruit, sugar and vegetable

	SFFQ		4DDD		Wilcoxon matched pairs	Spearman’s correlation	
	Median	(25th–75th)	Median	(25th–75th)	*P*	*r*_s_	*P*
Fat (g/day)	50	(33–77)	65	(49–87)	0.0013	0.4326	0.0010
Fruit (g/day)	120	(29–280)	125	(55–216)	0.2176	0.5123	< 0.0001
Sugars (g/day)	68	(31–111)	66	(45–94)	0.4977	0.4079	0.0020
Vegetables (g/day)	57	(29–120)	118	(86–181)	< 0.0001	0.3983	0.0026

**Fig. 1:**
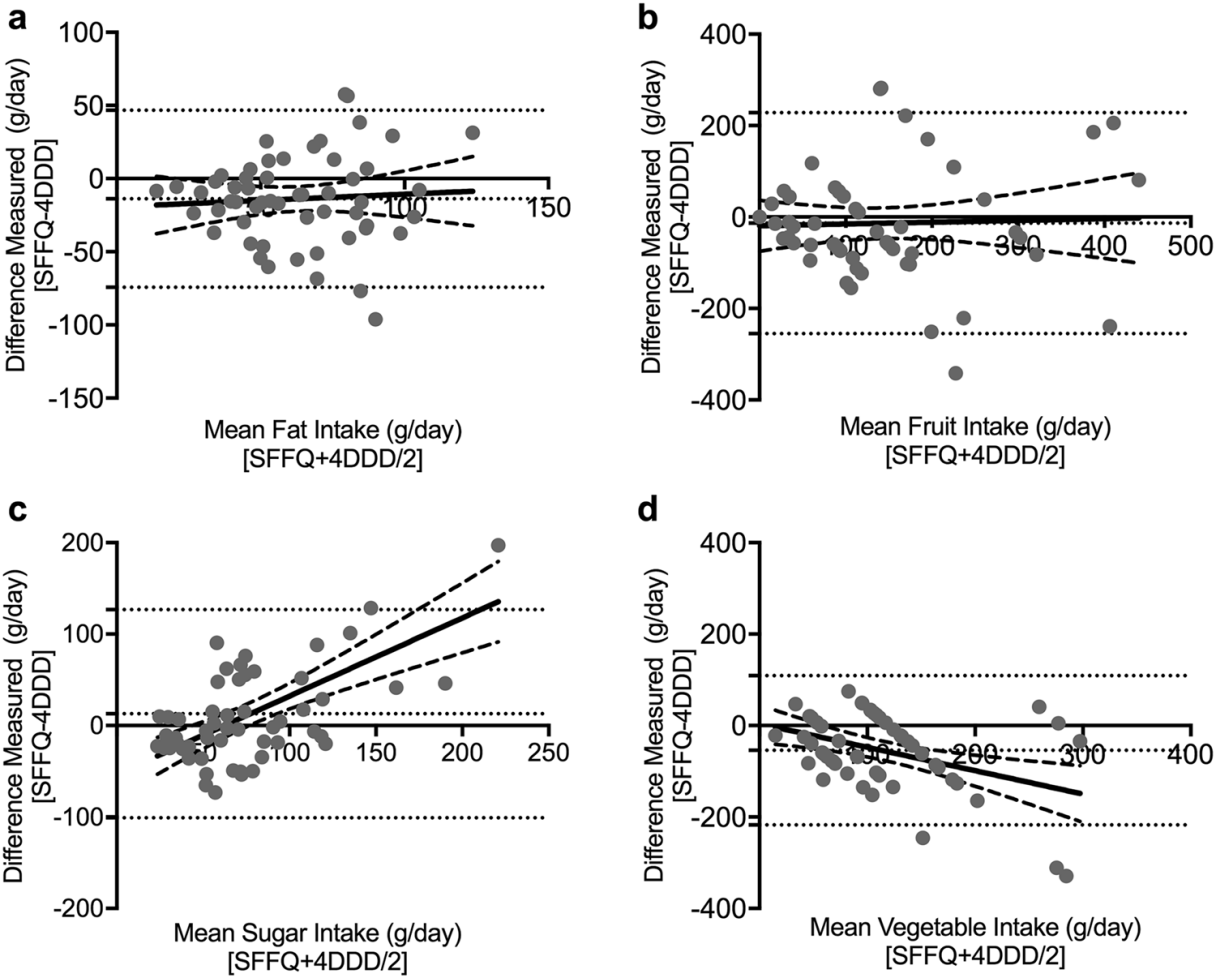
Bland Altman plots showing difference vs mean for the SFFQ and 4DDD measurements of: **a** sugar; **b** fat; **c** fruit; and **d** vegetable intakes. Bias and 95% limits of agreement shown in dotted horizontal lines. Linear regression and 95% confidence intervals are solid line and dashed lines, respectively

Bland Altman analysis of bias, plotting difference vs the mean to assess comparability of two methods of clinical measurement [38], in tandem with linear regression analysis was used to further assess agreement between the two methods, along with the distribution of bias (Fig. 1). Bias was minimal and evenly distributed for fat and fruit with the SFFQ underestimating fat (– 13.8 g/day; Fig. 1a) and slightly underestimating fruit (– 13.5 g/day; Fig. 1b) relative to the 4DDD. Although the overall bias for sugar was a relatively small overestimation (+ 12.9 g/day), regression showed dose-dependency with more skew evident at larger intakes (Fig. 1c). Larger underestimations were observed for the SFFQ measurement of vegetables (– 54.2 g/day; Fig. 1d), with regression suggesting greater underestimation at higher intakes (Fig. 1d). In general, agreement intervals were broad (Fig. 1).

## Discussion

This is the first study to characterise a cohort of adult Irish NAFLD patients and their dietary intakes. The majority of participants were obese and patients with biopsy-confirmed NASH were more likely to also be diagnosed with hypertension and type 2 diabetes. Dietary intakes were overwhelmingly consistent with the typical ‘Western diet’ associated with obesity, with high intakes of sugar, sodium, total and saturated fat; and substantially low intakes of vitamin D, omega-3 polyunsaturated fat and dietary fibre. Underreporting was common but in line with what is typically observed in population studies [32]. We observed no differences between the dietary intakes of patients diagnosed with simple steatosis (NAFL), and those diagnosed with steatohepatitis (NASH); rather, the vast majority of patients were consuming nutritionally poor diets, high in sugar, salt and saturated fat.

A primary objective was to develop and validate a minimally burdensome tool for assessing dietary intakes in NAFLD patients. Improvement in the clinical outcomes of NAFLD has been generally observed with a weight loss of 5–10% [6] and for this reason, dietary and lifestyle changes aimed at weight loss underpin clinical management guidelines for NAFLD [2, 3, 5]. While dietary assessment and dietetic management should ideally be made available to NAFLD patients in primary care and tertiary referral settings, this is not always possible. Therefore, brief dietary assessment tools, responsive to dietary changes, can allow health professionals to quickly identify areas of concern and help set and monitor food-based dietary goals for their patients [25]. Several tools exist for dietary management of cardiovascular disease and type 2 diabetes, and have successfully been used in clinical practice. However, the majority of these have been developed and evaluated in the US, and are not fully applicable to a European population [25].

To our knowledge, no such instrument (a brief dietary assessment tool, responsive to dietary changes) has been developed for NAFLD patients. As FFQs are easily administered in settings lacking formal dietetic support, and portion sizes are built into the questions reducing measurement error [15], we aimed to develop and validate an SFFQ for use with NAFLD patients. Typically, FFQs are routinely validated for a specific population being studied. Here, we adapted a concise 20-item SFFQ previously validated for conveniently assessing dietary quality in UK adults [18], considered a reasonable starting place for an inner-city Irish cohort of NAFLD patients, by adding additional questions from the EPIC-FFQ [16], emphasising questions related to refined carbohydrates, sugar-sweetened beverages, high-fat foods, and ultra-processed foods. The result was the 48-item SFFQ employed in this study.

The relative validity of our newly developed SFFQ was examined in relation to 4DDDs from 55 NAFLD patients, focusing on results for sugar, fat, fruit, and vegetables. This approach to validation of dietary assessment tools has been extensively utilised in the literature [18, 39–42]. Correlations between the two instruments for sugar, fat and fruit were highly significant and all nutrients had moderate correlation coefficients (0.4–0.51) very much in line with, or better than, reported correlations in comparable studies. For example, the correlation between fat measurements from the instruments here at 0.43 was much stronger than those observed by Cleghorn (0.22) [18], or Steinemann (0.37) [43]. The correlation (0.41) between the SFFQ and 4DDD sugar measurements, while not as strong as Yuan et al. (0.53) [26], was stronger than the 0.15 and 0.10 found by Cleghorn [18] and with the much longer (109 items) instrument of Tabacchi [44]. This justified our approach of adding further sugar-related questions to the concise set used in the Cleghorn 20-question tool. Bland–Altman plots were used to examine the average and the difference between the new SFFQ and the 4DDD instruments. This analysis demonstrated minimal bias in the SFFQ for fat, sugar and fruit intakes, but some underestimation by the SFFQ in vegetable intakes and some skewing at higher intakes for sugar and vegetables. Nonetheless, the methods were significantly correlated and showed broad comparability in the Bland Altman analysis.

The process of validating a new FFQ against a 4DDD is well-described as an acceptable, if flawed, validation method [45]. A 4DDD is an imperfect reference instrument, as measurement errors are inherent to this method of dietary assessment, and may result in correlated errors between both instruments [46, 47]. The potential for reactivity and/or recall biases are high when utilising most forms of dietary assessment instruments, particularly among overweight and obese individuals [15], which accounted for 96% of our cohort. While the potential for conscious energy restrictions to facilitate weight loss in some participants cannot be ruled out, the under-reporting of energy intakes observed in the 4DDDs here suggest some recall bias likely. Limitations of this work include the use of a sole patient cohort and risk of selection bias, which mean that it may not be generalizable to other populations. As previously discussed, FFQs are not instruments for the precise measurement of absolute energy or nutrient intakes [19, 20] but may be useful as adjunct clinical tools with acceptable ranking abilities [23]. Limitations in all concise tools surround choice of questions and we note this tool did not have questions specifically around healthful aspects of the Mediterranean diet such as nuts, seeds and specifically quantifying olive oil consumption, which will be addressed in future iterations.

Strengths of the study include the fact that it is the first characterisation of an Irish NAFLD cohort and their dietary intakes, the use of 4DDDs, the use of the comprehensive myfood24 database, and the researcher-led administration of the FFQ. While the gold-standard methodology for validating an FFQ is against biomarkers [15], these have their own limitations and were judged outside the scope of this study. Nonetheless, urinary sugars biomarkers, in particular, would be an interesting choice for future research; capturing two important factors at once: a form of calibration or calculating an adjustment equation for misreporting, as well as being a measure of dietary exposure of sugars [48]. Ideally, the examination of the SFFQ alongside urinary biomarkers in response to a dietary intervention would clarify the responsiveness of this instrument to dietary changes.

In the context of validating the novel SFFQ, 55 4DDDs from a cohort of Irish NAFLD patients were analysed and assessed. NAFLD in Ireland has been under-researched and these cross-sectional data are the first characterisation of an Irish NAFLD cohort and their dietary intakes. Dietary intakes were overwhelmingly consistent with the typical Western dietary patterns seen in other NAFLD populations around the world, with high-fat, high-sugar, high-sodium, low omega-3 polyunsaturated fat, and low fibre intakes dominating. A future aim is to further develop the SFFQ into an electronic tool capable of rapidly categorising a ‘NAFLD risk diet’ with the aim of providing effective key messages (e.g. decrease sugar intake), particularly in primary and tertiary care where resources are limited. While prevalence data for NAFLD in Ireland is lacking, population-based obesity data highlight a significant clinical burden, with 71% of older adults now classified as overweight and obese [49]. These figures agree with a Lancet report that shows Ireland is set to become the most obese nation in Europe by 2025 [50]. In adults with obesity and concomitant diseases such as type 2 diabetes, NAFLD incidence can increase to 69%. Additional factors, a review of which were outside the scope of this study but include Ireland’s higher incidence rates per capita of hereditary hemochromatosis, and higher rates per capita of binge-pattern alcohol consumption, justify concerns over a burgeoning liver disease epidemic. These data highlight the urgent need to identify those at risk of NAFLD in the community, and why primary and tertiary care teams require additional tools to rapidly and reliably assess dietary intakes that potentiate the disease state.

## Electronic supplementary material

Below is the link to the electronic supplementary material.

Supplementary material 1 (PDF 189 KB)

Supplementary material 2 (XLSX 171 KB)

## References

[1] Loomba R, Sanyal AJ (2013). The global NAFLD epidemic. Nat Rev Gastroenterol Hepatol.

[2] EASL-EASD-EASO (2016). EASL-EASD-EASO Clinical Practice Guidelines for the management of non-alcoholic fatty liver disease. J Hepatol.

[3] Chalasani N, Younossi Z, Lavine JE, Charlton M, Cusi K, Rinella M, Harrison SA, Brunt EM, Sanyal AJ (2018). The diagnosis and management of nonalcoholic fatty liver disease: practice guidance from the American Association for the study of liver diseases. Hepatology.

[4] Moore JB (2010). Non-alcoholic fatty liver disease: the hepatic consequence of obesity and the metabolic syndrome. Proc Nutr Soc.

[5] National Institute for Health and Care Excellence (2016) Non-alcoholic fatty liver disease: assessment and management. NICE guideline NG49. https://www.nice.org.uk/guidance/ng49/evidence/full-guideline-pdf-2548213310 27441333

[6] Kenneally S, Sier JH, Moore JB (2017). Efficacy of dietary and physical activity intervention in non-alcoholic fatty liver disease: a systematic review. BMJ Open Gastroenterol.

[7] George ES, Forsyth A, Itsiopoulos C, Nicoll AJ, Ryan M, Sood S, Roberts SK, Tierney AC (2018). Practical dietary recommendations for the prevention and management of nonalcoholic fatty liver disease in adults. Adv Nutr.

[8] Pownall HJ, Bray GA, Wagenknecht LE, Walkup MP, Heshka S, Hubbard VS, Hill J, Kahn SE, Nathan DM, Schwartz AV, Johnson KC (2015). Changes in body composition over 8 years in a randomized trial of a lifestyle intervention: the look AHEAD study. Obesity.

[9] Eslamparast T, Tandon P, Raman M (2017). Dietary composition independent of weight loss in the management of non-alcoholic fatty liver disease. Nutrients.

[10] Properzi C, O’Sullivan TA, Sherriff JL, Ching HL, Jeffrey GP, Buckley RF, Tibballs J, MacQuillan GC, Garas G, Adams LA (2018). Ad libitum Mediterranean and low-fat diets both significantly reduce hepatic steatosis: a randomized controlled trial. Hepatology.

[11] Rees K, Hartley L, Flowers N, Clarke A, Hooper L, Thorogood M, Stranges S (2013). ‘Mediterranean’ dietary pattern for the primary prevention of cardiovascular disease. Cochrane Database Syst Rev.

[12] Rosato V, Temple NJ, La Vecchia C, Castellan G, Tavani A, Guercio V (2017). Mediterranean diet and cardiovascular disease: a systematic review and meta-analysis of observational studies. Eur J Nutr.

[13] Garcia M, Bihuniak JD, Shook J, Kenny A, Kerstetter J, Huedo-Medina TB (2016). The effect of the traditional mediterranean-style diet on metabolic risk factors: a meta-analysis. Nutrients.

[14] Rebro SM, Patterson RE, Kristal AR, Cheney CL (1998). The effect of keeping food records on eating patterns. J Am Diet Assoc.

[15] Thompson FE, Kirkpatrick SI, Subar AF, Reedy J, Schap TE, Wilson MM, Krebs-Smith SM (2015). The National Cancer Institute’s dietary assessment primer: a resource for diet research. J Acad Nutr Diet.

[16] Bingham SA, Welch AA, McTaggart A, Mulligan AA, Runswick SA, Luben R, Oakes S, Khaw KT, Wareham N, Day NE (2001). Nutritional methods in the European Prospective Investigation of Cancer in Norfolk. Public Health Nutr.

[17] Cade JE, Burley VJ, Greenwood DC (2004). The UK Women’s Cohort Study: comparison of vegetarians, fish-eaters and meat-eaters. Public Health Nutr.

[18] Cleghorn CL, Harrison RA, Ransley JK, Wilkinson S, Thomas J, Cade JE (2016). Can a dietary quality score derived from a short-form FFQ assess dietary quality in UK adult population surveys?. Public Health Nutr.

[19] Kroke A, Klipstein-Grobusch K, Voss S, Moseneder J, Thielecke F, Noack R, Boeing H (1999). Validation of a self-administered food-frequency questionnaire administered in the European Prospective Investigation into Cancer and Nutrition (EPIC) Study: comparison of energy, protein, and macronutrient intakes estimated with the doubly labeled water, urinary nitrogen, and repeated 24-h dietary recall methods. Am J Clin Nutr.

[20] Schatzkin A, Kipnis V, Carroll RJ, Midthune D, Subar AF, Bingham S, Schoeller DA, Troiano RP, Freedman LS (2003). A comparison of a food frequency questionnaire with a 24-hour recall for use in an epidemiological cohort study: results from the biomarker-based Observing Protein and Energy Nutrition (OPEN) study. Int J Epidemiol.

[21] Hebert JR, Hurley TG, Steck SE, Miller DR, Tabung FK, Peterson KE, Kushi LH, Frongillo EA (2014). Considering the value of dietary assessment data in informing nutrition-related health policy. Adv Nutr.

[22] Cade JE (2017). Measuring diet in the 21st century: use of new technologies. Proc Nutr Soc.

[23] Medin AC, Carlsen MH, Hambly C, Speakman JR, Strohmaier S, Andersen LF (2017). The validity of a web-based FFQ assessed by doubly labelled water and multiple 24-h recalls. Brit J Nutr.

[24] FAO (2018) Dietary assessment: a resource guide to method selection and application in low resource settings. Rome. http://www.fao.org/3/i9940en/I9940EN.pdf

[25] England CY, Andrews RC, Jago R, Thompson JL (2015). A systematic review of brief dietary questionnaires suitable for clinical use in the prevention and management of obesity, cardiovascular disease and type 2 diabetes. Eur J Clin Nutr.

[26] Food Safety Authority of Ireland (2011) Scientific recommendations for healthy eating guidelines in Ireland. https://www.fsai.ie/WorkArea/DownloadAsset.aspx?id=16765

[27] Carter MC, Albar SA, Morris MA, Mulla UZ, Hancock N, Evans CE, Alwan NA, Greenwood DC, Hardie LJ, Frost GS, Wark PA, Cade JE (2015). Development of a UK Online 24-h dietary assessment tool: myfood24. Nutrients.

[28] Public Health England (2015) McCance and Widdowson’s the composition of foods integrated dataset. https://www.gov.uk/government/publications/composition-of-foods-integrated-dataset-cofid

[29] Carter MC, Hancock N, Albar SA, Brown H, Greenwood DC, Hardie LJ, Frost GS, Wark PA, Cade JE (2016). Development of a new branded UK food composition database for an online dietary assessment tool. Nutrients.

[30] Mendez MA, Popkin BM, Buckland G, Schroder H, Amiano P, Barricarte A, Huerta JM, Quiros JR, Sanchez MJ, Gonzalez CA (2011). Alternative methods of accounting for underreporting and overreporting when measuring dietary intake-obesity relations. Am J Epidemiol.

[31] Huang TT, Roberts SB, Howarth NC, McCrory MA (2005). Effect of screening out implausible energy intake reports on relationships between diet and BMI. Obes Res.

[32] Murakami K, Livingstone MB (2015). Prevalence and characteristics of misreporting of energy intake in US adults: NHANES 2003–2012. Brit J Nutr.

[33] Yuan C, Spiegelman D, Rimm EB, Rosner BA, Stampfer MJ, Barnett JB, Chavarro JE, Subar AF, Sampson LK, Willett WC (2017). Validity of a dietary questionnaire assessed by comparison with multiple weighed dietary records or 24-hour recalls. Am J Epidemiol.

[34] Moore JB (2019) Non-alcoholic fatty liver disease short food frequency questionnaire patient information leaflet [Dataset]. 10.5518/478

[35] Lombard MJ, Steyn NP, Charlton KE, Senekal M (2015). Application and interpretation of multiple statistical tests to evaluate validity of dietary intake assessment methods. Nutrition J.

[36] Moore JB, Fielding BA (2016). Sugar and metabolic health: is there still a debate?. Curr Opin Clin Nutr Metab Care.

[37] Food Safety Authority of Ireland (1999) Recommended dietary allowances for Ireland. http://hdl.handle.net/10147/44808

[38] Giavarina D (2015). understanding bland altman analysis. Biochem Med.

[39] Laviolle B, Froger-Bompas C, Guillo P, Sevestre A, Letellier C, Pouchard M, Daubert JC, Paillard F (2005). Relative validity and reproducibility of a 14-item semi-quantitative food frequency questionnaire for cardiovascular prevention. Eur J Cardiovasc Prev Rehabil.

[40] Fernandez-Ballart JD, Pinol JL, Zazpe I, Corella D, Carrasco P, Toledo E, Perez-Bauer M, Martinez-Gonzalez MA, Salas-Salvado J, Martin-Moreno JM (2010). Relative validity of a semi-quantitative food-frequency questionnaire in an elderly Mediterranean population of Spain. Br J Nutr.

[41] Bentzen SM, Knudsen VK, Christiensen T, Ewers B (2016). Relative validity of a web-based food frequency questionnaire for patients with type 1 and type 2 diabetes in Denmark. Nutr Diabetes.

[42] Villena-Esponera MP, Moreno-Rojas R, Romero-Saldana M, Molina-Recio G (2017). Validation of a food frequency questionnaire for the indigenous epera-siapidara people in Ecuador. Nutr Hosp.

[43] Steinemann N, Grize L, Ziesemer K, Kauf P, Probst-Hensch N, Brombach C (2017). Relative validation of a food frequency questionnaire to estimate food intake in an adult population. Food Nutr Res.

[44] Tabacchi G, Filippi AR, Breda J, Censi L, Amodio E, Napoli G, Bianco A, Jemni M, Firenze A, Mammina C (2015). Comparative validity of the ASSO-Food Frequency Questionnaire for the web-based assessment of food and nutrients intake in adolescents. Food Nutr Res.

[45] Devenish G, Mukhtar A, Begley A, Do L, Scott J (2017). Development and relative validity of a food frequency questionnaire to assess intakes of total and free sugars in australian toddlers. Int J Environ Res Public Health.

[46] Willett WC, Lenart E, Willett WC (2013). Reproducibility and validity of food-frequency questionnaires. Nutritional epidemiology.

[47] Kipnis V, Subar AF, Midthune D, Freedman LS, Ballard-Barbash R, Troiano RP, Bingham S, Schoeller DA, Schatzkin A, Carroll RJ (2003). Structure of dietary measurement error: results of the OPEN biomarker study. Am J Epidemiol.

[48] Tasevska N (2015). Urinary sugars—a biomarker of total sugars intake. Nutrients.

[49] Nolan A, O’Regan C, Dooley C, Wallace D, Hever A, Cronin H, Hudson E (2014). The over 50 s in a changing ireland: economic circumstances, health and wellbeing.

[50] NCD-RisC NCD, Risk Factor C (2016). Trends in adult body-mass index in 200 countries from 1975 to 2014: a pooled analysis of 1698 population-based measurement studies with 19.2 million participants. Lancet.

