# Supplementary material for: Development and relative validation of a short food frequency questionnaire for assessing dietary intakes of non-alcoholic fatty liver disease patients
Source: Eur J Nutr. 2019 Feb 25;59(2):571–80. doi: 10.1007/s00394-019-01926-5 (PMC7058564; doi:10.1007/s00394-019-01926-5)
Supplement: Supplementary file 1 — Supplementary material 1 (PDF 189 KB) [file 394_2019_1926_MOESM1_ESM.pdf]

The following questions ask about some foods & drinks you might have during a 'typical' week, over the past month or so. Do not be concerned if some things you eat or drink are not mentioned.

Please tick how often you eat at least ONE portion of the following foods & drinks: (a portion includes: a handful of grapes, an orange, a serving of carrots, a side salad, a slice of bread, a glass of pop).

*(Please only put one tick, but answer **EVERY** line)*

1. Carbohydrates

		Rarely or never	Less than 1 a week	Once a week	2-3 times a week	4-6 times a week	1-2 times a day	3-4 times a day	5+ a day
1.1	Fruit - fresh and frozen	☐	☐	☐	☐	☐	☐	☐	☐
1.2	Fruit - tinned in syrup, dried	☐	☐	☐	☐	☐	☐	☐	☐
1.3	Fruit juice (not cordial or squash)	☐	☐	☐	☐	☐	☐	☐	☐
1.4	Salad (not garnish added to sandwiches)	☐	☐	☐	☐	☐	☐	☐	☐
1.5	Vegetables (tinned/frozen/fresh but not potatoes)	☐	☐	☐	☐	☐	☐	☐	☐
1.6	Chips/fried potatoes	☐	☐	☐	☐	☐	☐	☐	☐
1.7	Potatoes, boiled, steamed, baked	☐	☐	☐	☐	☐	☐	☐	☐
1.8	Beans or pulses like baked beans, chick peas, dahl	☐	☐	☐	☐	☐	☐	☐	☐
1.9	White bread, rolls	☐	☐	☐	☐	☐	☐	☐	☐
1.10	Brown bread, rolls	☐	☐	☐	☐	☐	☐	☐	☐
1.11	Fibre-rich breakfast cereal, like Weetabix, Fruit 'n' Fibre, Porridge, Muesli	☐	☐	☐	☐	☐	☐	☐	☐

© University of Leeds (2018); DOI: http://doi.org/10.5518/477
This work is licensed under a Creative Commons (CC-BY 4.0)

		Rarely or never	Less than 1 a week	Once a week	2-3 times a week	4-6 times a week	1-2 times a day	3-4 times a day	5+ a day
1.12	Breakfast Cereals (cornflakes, rice krispies)	☐	☐	☐	☐	☐	☐	☐	☐
1.13	White rice, white pasta	☐	☐	☐	☐	☐	☐	☐	☐
1.14	Brown rice, brown pasta	☐	☐	☐	☐	☐	☐	☐	☐
1.15	Fizzy soft drinks e.g. coca cola, lemonade	☐	☐	☐	☐	☐	☐	☐	☐
1.16	Low calorie or diet fizzy drinks, or squash, or cordial	☐	☐	☐	☐	☐	☐	☐	☐
1.17	Sweet biscuits, cakes, chocolate, sweets	☐	☐	☐	☐	☐	☐	☐	☐
1.18	Ice cream/choc ice	☐	☐	☐	☐	☐	☐	☐	☐
1.19	Sugar added to tea/coffee/cereal (teaspoon)	☐	☐	☐	☐	☐	☐	☐	☐
1.20	Crisps / savoury snacks	☐	☐	☐	☐	☐	☐	☐	☐
1.21	Tea (cup)	☐	☐	☐	☐	☐	☐	☐	☐
1.22	Coffee, instant or ground (cup)	☐	☐	☐	☐	☐	☐	☐	☐
1.23	Coffee drink, made with flavoured syrups, e.g hazelnut latte, frappuccino, mocha	☐	☐	☐	☐	☐	☐	☐	☐
1.24	Cocoa, hot chocolate (cup)	☐	☐	☐	☐	☐	☐	☐	☐

© University of Leeds (2018); DOI: http://doi.org/10.5518/477
This work is licensed under a Creative Commons (CC-BY 4.0)

2. Protein

		Rarely or never	Less than 1 a week	Once a week	2-3 times a week	4-6 times a week	7+ times a week
**Whole meats:**							
2.1	Beef, Lamb, Pork, Ham - steaks, roasts, joints, mince or chops	☐	☐	☐	☐	☐	☐
2.2	Chicken or Turkey – steaks, roasts, joints, mince or portions (not in batter or breadcrumbs)	☐	☐	☐	☐	☐	☐
**Processed meats/meat:**							
2.3	Sausages, bacon, corned beef, meat pies/pasties, burgers	☐	☐	☐	☐	☐	☐
2.4	Chicken or Turkey - nuggets/twizzlers, turkey burgers, chicken pies, or in batter or breadcrumbs	☐	☐	☐	☐	☐	☐
**Fish:**							
2.5	White fish in batter or breadcrumbs – like 'fish 'n' chips'	☐	☐	☐	☐	☐	☐
2.6	White fish not in batter or breadcrumbs	☐	☐	☐	☐	☐	☐
2.7	Oily fish – like herrings, sardines, salmon, trout, mackerel, fresh tuna (not tinned tuna)	☐	☐	☐	☐	☐	☐

3. Fat

		Rarely or never	Less than 1 a week	Once a week	2-3 times a week	4-6 times a week	7+ times a week
**Dairy Products:**							
3.1	Low fat yoghurt, fromage frais, flavoured yoghurt (125g carton)	☐	☐	☐	☐	☐	☐
3.2	Full fat, Greek yoghurt, or natural yoghurt (125g carton)	☐	☐	☐	☐	☐	☐
3.3	Cheese, e.g. cheddar, brie, edam (medium serving)	☐	☐	☐	☐	☐	☐
3.4	Cottage cheese, low fat soft cheese (medium serving)	☐	☐	☐	☐	☐	☐

© University of Leeds (2018); DOI: http://doi.org/10.5518/477
This work is licensed under a Creative Commons (CC-BY 4.0)

		Rarely or never	Less than 1 a week	Once a week	2-3 times a week	4-6 times a week	7+ times a week
**Spreads:**							
3.5	Butter (teaspoon)	☐	☐	☐	☐	☐	☐
3.6	Block margarine e.g. stork (teaspoon)	☐	☐	☐	☐	☐	☐
3.7	Polyunsaturated margarine (tub) eg Flora, Dairygold, sunflower spread (teaspoon)	☐	☐	☐	☐	☐	☐
3.8	Other soft margarine, dairy spreads (tub) e.g Low Low, Connaught Gold (teaspoon)	☐	☐	☐	☐	☐	☐
3.9	Salad cream/mayonnaise	☐	☐	☐	☐	☐	☐

4. Are there any other foods which you ate more than once a week?

Yes☐ No☐ (If yes, please list below)

Food	Usual Serving	No of times eaten each week

5. On average, how many portions of FRUIT do you eat a day?

____

(examples include: a handful of grapes, an orange, a handful of dried fruits)

6. On average, how many portions of VEGETABLES do you eat a day?

____

(examples include: 3 heaped tablespoons of carrots, a side salad, 2 spears of broccoli)

7. What milk do you usually use or drink, such as in hot & cold drinks or on cereal?

(including tea, coffee, hot milk, milk shakes, or on cereal)

Whole/ Full-fat milk	☐	Semi-skimmed milk	☐
Skimmed milk	☐	Rarely/never use milk	☐
Other (please specify)			

8. How often did you eat takeaway foods such as pizza/curries/fish and chips?

Daily	☐	1-3 times a week	☐
4-6 times a week	☐	Less than once a week	☐
Never	☐		

© University of Leeds (2018); DOI: http://doi.org/10.5518/477
This work is licensed under a Creative Commons (CC-BY 4.0)

9. What type of fat did you most often use for frying, roasting, grilling etc?

Butter	☐	Lard/dripping	☐
Vegetable oil<br>please specify, e.g. corn, sunflower	☐	Solid vegetable fat (cisco, stork)	☐
Margarine	☐	None	☐

10. How often did you eat food that was fried at home?

Daily	☐	1-3 times a week	☐
4-6 times a week	☐	Less than once a week	☐
Never	☐		

11. On average, how much alcohol do you drink over a complete seven day week?

One standard drink is 10g of alcohol, which equals 1 small glass of wine (100ml of 12.5%ABV), half a pint of beer, lager, or stout (284ml), a pub measure of spirits (35.5ml). See photos for guide

I rarely/never drink alcohol	☐	Less than 11 standard drinks	☐
Between 11 and 17 standard drinks	☐	More than 17 standard drinks	☐

Thank you

Courtesy of Drink Aware www.drinkaware.ie

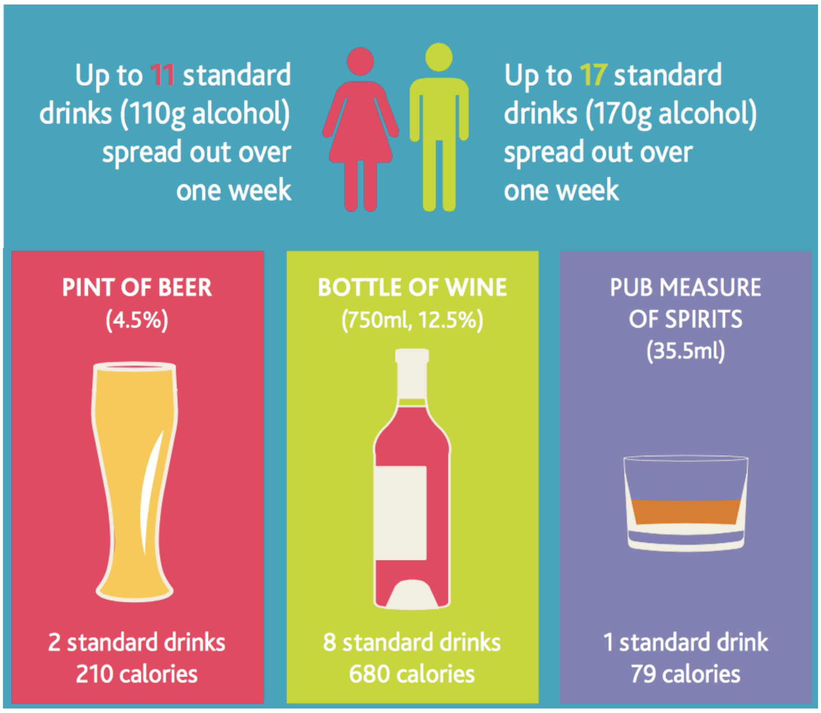

© University of Leeds (2018); DOI: http://doi.org/10.5518/477
This work is licensed under a Creative Commons (CC-BY 4.0)
